# In Situ Detection of Endogenous HIV Activation by Dynamic Nuclear Polarization NMR and Flow Cytometry

**DOI:** 10.3390/ijms21134649

**Published:** 2020-06-30

**Authors:** Sarah A. Overall, Lauren E. Price, Brice J. Albert, Chukun Gao, Nicholas Alaniva, Patrick T. Judge, Erika L. Sesti, Paul A. Wender, George B. Kyei, Alexander B. Barnes

**Affiliations:** 1 Laboratory of Physical Chemistry, ETH Zurich, 8093 Zurich, Switzerland; lauren.price@phys.chem.ethz.ch (L.E.P.); chukun.gao@phys.chem.ethz.ch (C.G.); nicholas.alaniva@phys.chem.ethz.ch (N.A.); 2 Department of Chemistry, Washington University in St. Louis, St. Louis, MO 63130, USA; b.albert@wustl.edu.com (B.J.A.); judgept@wustl.edu (P.T.J.); elsesti@wustl.edu (E.L.S.); 3 Department of Biochemistry, Biophysics and Structural Biology, Washington University in St. Louis, St. Louis, MO 63110, USA; 4 Departments of Chemistry and Chemical and Systems Biology, Stanford University, Stanford, CA 94305, USA; 5 Department of Medicine, Washington University School of Medicine in St. Louis, St. Louis, MO 63110, USA; 6 Noguchi Memorial Institute for Medical Research, University of Ghana, Accra 00233, Ghana

**Keywords:** HIV, viral latency, dynamic nuclear polarization, in-cell NMR, solid-state NMR

## Abstract

We demonstrate for the first time in-cell dynamic nuclear polarization (DNP) in conjunction with flow cytometry sorting to address the cellular heterogeneity of in-cell samples. Utilizing a green fluorescent protein (GFP) reporter of HIV reactivation, we correlate increased ^15^N resonance intensity with cytokine-driven HIV reactivation in a human cell line model of HIV latency. As few as 10% GFP+ cells could be detected by DNP nuclear magnetic resonance (NMR). The inclusion of flow cytometric sorting of GFP+ cells prior to analysis by DNP-NMR further boosted signal detection through increased cellular homogeneity with respect to GFP expression. As few as 3.6 million ^15^N-labeled GFP+ cells could be readily detected with DNP-NMR. Importantly, cell sorting allowed for the comparison of cytokine-treated GFP+ and GFP− cells in a batch-consistent way. This provides an avenue for normalizing NMR spectral contributions from background cellular processes following treatment with cellular modulators. We also demonstrate the remarkable stability of AMUPol (a nitroxide biradical) in Jurkat T cells and achieved in-cell enhancements of 46 with 10 mM AMUPol, providing an excellent model system for further in-cell DNP-NMR studies. This represents an important contribution to improving in-cell methods for the study of endogenously expressed proteins by DNP-NMR.

## 1. Introduction

Nuclear magnetic resonance (NMR) is an exceptional method for studying molecular structure and dynamics within complex biological systems including lipid-bilayers and membrane proteins [1], fibrils [2,3,4] and cell walls [5,6]. Viral systems [7,8,9,10] and intact cells [11,12,13,14,15,16] are particularly accessible to solid-state NMR due to its independence from molecular correlation times, and therefore particle size. However, the inherently low sensitivity of solid-state NMR due to the low Boltzmann polarization of nuclei poses a significant challenge. This is exacerbated by dipolar-mediated line broadening, even under magic angle spinning (MAS) conditions. Solid-state NMR typically requires high sample concentrations and homogeneity in order to achieve sufficient sensitivity. This is a particular challenge for heterogenous, amorphous samples in which individual spins of interest may be diluted.

MAS dynamic nuclear polarization (DNP) solid-state NMR leverages the high gyromagnetic ratio of electron spins to transfer high spin polarization from a free unpaired electron (in the form of a stable radical) to the nuclei of interest using microwave irradiation. This greatly enhances nuclear spin sensitivity. The theoretical limit for ^1^H enhancements is 660-fold, with experimental enhancements of up to 515 demonstrated on model systems [17]. The increased signal intensity afforded by DNP has allowed the observation and characterization of previously unknown conformational states of α-synuclein [18], HIV protein assemblies [19] and G protein coupled receptors (GPCRs) [20]. DNP solid-state NMR has also proved invaluable for the study of membrane proteins in which protein spins are significantly diluted by the supporting lipid structures [21,22] and even allowed the characterization of membrane proteins in native cellular membranes [23].

The dilution of individual proteins within heterogenous cellular structures pushes the sensitivity limits of DNP. Heterogeneity between cells is an additional challenge for in-cell NMR studies, especially primary cells. Inducible protein expression systems also show variability in expression levels which may give rise to aggregate formation or artifactual protein–protein interactions depending on the in-cell concentration. Not only does this further complicate the NMR spectra acquired on such samples, reducing resolution and thus interpretability, but it further dilutes the spins of interest as the net signal intensity is divided between all the conformers, reducing sensitivity.

Recently, DNP has been applied to these challenging cellular systems. We previously reported the application of MAS-DNP to cellular samples, obtaining 46-fold enhancements of ^15^N resonances in intact human embryonic kidney cell 293 (HEK293) cells using the conventional DNP radical AMUPol [24]. AMUPol is a highly water soluble, stable bi-nitroxide radical which has become the standard radical used to perform DNP [25]. We achieved even greater in-cell enhancements using a trimodal DNP reagent in which the radical TOTAPOL was tethered to a fluorochrome-conjugated cell-penetrating peptide. This unique reagent additionally provided control over the cellular localization of the DNP radical in addition to the direct assessment of radical localization by microscopy [24]. Other laboratories have also demonstrated the use of radical tagged biomolecules, including the antimicrobial peptides used to observe the membrane-associated resonances in intact bacteria [26]. Petzold and colleagues used DNP-NMR to observe antisense oligonucleotide drugs delivered to HEK 293T cells [27]. Isotopically labeled ubiquitin delivered into HeLa cells have also been observed by DNP using the radical PyPOL-TMR, a fluorescent variant of AMUPol [28]. Furthermore, selective ^13^C labeling has been combined with in-cell DNP to observe overexpressed, endogenous cytochrome b5 in intact *E. coli* cells [29].

There is little structural information that addresses latent HIV reactivation pathways in T-cells, despite the success of DNP-NMR in revealing important conformational changes in HIV capsid proteins critical for the maturation of viral particles [19]. Latent HIV infection remains a significant barrier to curing infected patients [30,31,32,33]. Current anti-retroviral therapy effectively reduces plasma viremia [34,35,36] but fails to eliminate latently infected cells, allowing the infection to persist for life. Many strategies aimed at targeting this latent reservoir of infected cells include small molecule latency-reversing agents (LRAs) [35] such as the PKC modulator bryostatin [36,37,38], and histone deacetylase (HDAC) inhibitors [39,40]. A better understanding of the structure and function of these small molecules and their targets within the cellular context would permit the design and synthesis of more potent and less toxic latency modulators [1,36,37,41].

Here, we demonstrated an in-cell DNP-NMR model to observe TNFα-mediated endogenous HIV reactivation in Jurkat T cells. We correlated GFP expression with increased ^15^N-amide resonance intensities, demonstrating the feasibility of using DNP-NMR to monitor endogenous HIV reactivation. We further improved the sensitivity of detection by employing fluorescence-activated cell sorting (FACS) to greatly enhance the homogeneity of the cell population. Furthermore, we demonstrated the potential of Jurkat T cells as a model system for in-cell studies due to slow AMUPol reduction in this cell line.

## 2. Results and Discussion

### 2.1. AMUPol is Stable in JLat T Cells

**Figure 1:**
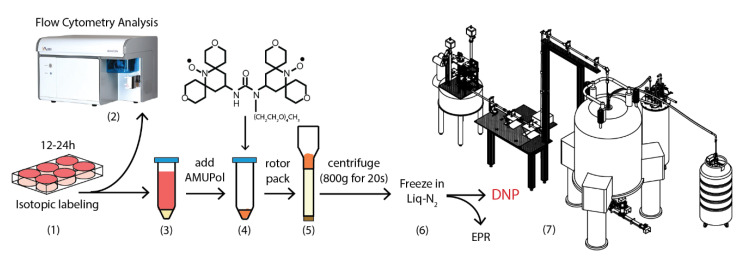
Preparation of the Jurkat T cells for dynamic nuclear polarization (DNP)-NMR. (1) Jurkat T cells are cultured for 12–24 h in isotopically labeled by the complete Roswell Park Memorial Institute (cRPMI). (2) A small aliquot of cells is taken for flow cytometric analysis. (3) The rest are pelleted by centrifugation and washed with phosphate buffered saline. (4) AMUPol and cryoprotectant are added directly to the cell pellet. (5) The cells are centrifuged for 20 s into a 3.2 mm ZrO_2_ rotor or electron paramagnetic resonance (EPR) tube. (6) Packed rotors or EPR tubes are immediately frozen in liquid nitrogen. (7) DNP solid-state NMR and EPR analysis of Jurkat cells is then carried out. See Materials and Methods for additional details.

To develop a model system for studying endogenously produced HIV by NMR, we utilized JLat 10.6 cells which are a variant of the Jurkat T cell line [42]. The HIV genome has been integrated into the host cell genome and provides a valuable model for studying the HIV reactivation from latency [42,43,44]. These cells produce near complete virions (but which lack the *nef* and *env* genes) in an NFκB-dependent manner [42,45]. We prepared ^15^N-labeled JLat cells for DNP-NMR by resuspension in 10% DMSO/90% phosphate buffered saline (PBS) and AMUPol to a final concentration of 10 mM (Figure 1). Rotor-packed cell samples were immediately frozen in liquid nitrogen to prevent radical reduction. We consistently achieved packing times of less than 2 min between the addition of the radical and the freezing in liquid nitrogen.

To ensure the maximum consistency between the rotors in terms of cell mass per rotor, specially designed Teflon funnels were used in which the neck of the funnel occupied 26 μL of the rotor during the packing, leaving a filling volume of 36 μL for the cell samples (Supplementary Materials SI-1), while allowing sufficient space for the insertion of the rotor drive tip.

**Figure 2:**
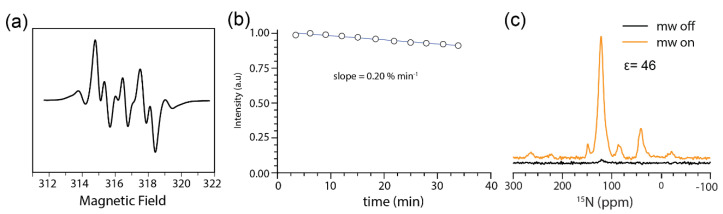
EPR analysis of AMPUPol stability in non-stimulated JLat T cells. (**a**) EPR spectra of AMUPol in JLat T cells at 9.4 GHz. (**b**) Integrated area of EPR signal over time. JLat cells were prepared with 10 mM AMUPol in 10% DMSO/90% phosphate buffered saline (PBS) and analyzed at room temperature. Data shown is one of two independent experiments. The average decay is 0.20% min^−1^ ± 0.08 s.d. (**c**) Comparison of microwave-on and -off spectra of selectively ^15^N- labeled JLat cells measured at 90 K. Spectra were acquired at 7.05 T with 197.674 GHz microwave irradiation, 4.5 kHz magic angle spinning (MAS) and 2048 transients.

The stability of AMUPol in the JLat cells was assessed by EPR spectroscopy at room temperature. After 30 min, we observed an 8% reduction in the integrated area of the AMUPol EPR spectrum, giving a decay rate of 0.20% min^−1^ ± 0.08, which is remarkably slow, indicating a relatively stable environment for DNP (Figure 2a,b). A DNP enhancement of 46 was measured at 300 MHz for the ^15^N amide resonances of JLat cells prepared in the same way (Figure 2c). We have previously reported a 30% reduction in the integrated area of the AMUPol EPR spectrum after 30 min in HEK293 cells [24]. In addition, recently reported decay rates in bacteria are even greater with a 75% reduction of a paramagnetic radical within 10 min [46]. *Xenopus laevis* oocytes, a common model system for in-cell structural studies, show a range of radical lifetimes from 3.8 min (TEMPOL radical) [47] to over 1 h (MTSSL radical) [48]. It is unclear why AMUPol is so stable in JLat T cells. The net redox potential of JLat cells might be different from other cell types previously studied with AMUPol as redox processes are the greatest source of DNP radical instability [46]. Regardless of the mechanism, the data suggest that JLat T cells are a good model cell type for DNP studies.

### 2.2. Detection of Endogenously Expressed HIV Proteins by DNP-MAS NMR

**Figure 3:**
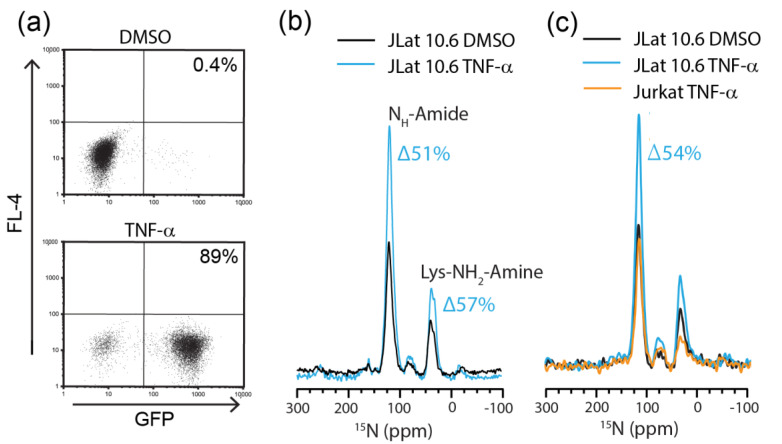
Characterization of HIV production in JLat 10.6 cells by MAS-DNP and flow cytometry. (**a**) Flow cytometry of the GFP expression by JLat 10.6 cells. Percentages in the plots indicate the proportion of total live cells that are GFP+ (bottom right quadrant). (**b**) ^15^N cross-polarization magic angle spinning (CPMAS)-DNP spectra of 1% DMSO (black line)- or TNF-α (blue line)-treated ^15^N-labeled JLat 10.6 cells. (**c**) ^15^N CPMAS-DNP spectra of Jurkat (orange line) and JLat 10.6 cells stimulated with TNFα (blue line) or the DMSO control (black line). Spectra were acquired at 7.05 T and 95 K with 197.674 GHz microwave irradiation, 4.5 kHz MAS and 2048 transients. (**b**,**c**) are independent experiments.

We used two distinct JLat T cell lines in which the HIV genome was latently integrated and commercially available [42]. JLat 10.6 and JLat 9.2 cells differ only in the chromosomal region in which the HIV genome is integrated [43]. This results in varying HIV activation between the two cell lines upon stimulation, where the JLat 10.6 cell line is easier to activate. These cells are infected with a recombinant HIV in which the *nef* gene has been replaced with the reporter green fluorescent protein (GFP), in addition to a mutation in *env* that prevents the formation of infectious virions [42]. Therefore, JLat cell lines can safely be assayed and analyzed in a standard biosafety 2 laboratory. Both cell lines exhibit low basal HIV activation, as monitored by GFP fluorescence (Figure 3a), consistent with published results [43]. The expression of GFP and HIV genes are both driven by the same promoter and therefore results in the coexpression of GFP with Gag-pol/vpu/vif/vpr/rev and tat polypeptides. GFP fluorescence intensity has been shown to directly correlate with HIV expression levels in this cell line by Western blotting for the Gag protein [49,50]. The addition of T cell activators induces HIV and GFP production in JLat cells [38,51].

To detect endogenously expressed HIV proteins by NMR, JLat 10.6 cells were cultured for the exponential growth phase in unlabeled media. The cells were then transferred to selectively ^15^N-enriched media and stimulated with the T cell activator, TNF-α for 12 h. HIV activation was assessed by flow cytometry. The stimulation with TNF-α resulted in 89% of JLat 10.6 cells upregulating GFP compared to 0.4% of the cells treated with 1% DMSO (vehicle control) (Figure 3a). The analysis of these same cells by DNP-NMR showed that the TNF-α treatment increased ^15^N amide resonance intensities by 51% and amine resonances by 57%, compared to the DMSO controls (Figure 3b). Thus, the increased signal intensity is likely attributed to endogenous HIV and GFP expression. The comparison of the amino acid sequences of all the HIV proteins produced with that of GFP indicates that under our labeling conditions, 89% of the ^15^N signal intensity can be attributed to HIV proteins and 11% to GFP, assuming all other things are equal (Supplementary Materials SI-2). Due to the well documented and published overexpression of HIV-related proteins in this cell line (in excess of 85,000 pg/mL) [42] we expect a large proportion of the ^15^N signal to come from HIV proteins through overexpression, although all other proteins produced during the labeling period will also be labeled.

TNF-α is known to increase protein expression in stimulated T cells, predominantly in the form of cytokine production, and might partially account for the observed increase in ^15^N resonance intensity [51]. We addressed this possibility by comparing the TNF-α-stimulated JLat 10.6 cells with stimulated Jurkat T cells, which do not contain any HIV DNA but are equally responsive to TNFα stimulation. The comparison of ^15^N amide resonance intensities showed a 54% increase in signal intensity following the stimulation of JLat 10.6 cells compared to the stimulated Jurkat cells (Figure 3c). Thus, the increase in ^15^N resonance intensity cannot be accounted for by cytokine production, which was expected as cytokines are secreted proteins and they are washed away in the sample preparation process. This is a clear demonstration of endogenous HIV and GFP detection by DNP solid-state NMR. Furthermore, the proportion of GFP+ cells also correlates reasonably well with increases in ^15^N signal intensity.

Increases in the ^15^N resonance intensity due to the bulk activation of JLat 10.6 cells were readily detectable. However, the proportion of latently infected T cells in vivo is typically very low [52]. In order to assess the feasibility of using MAS-DNP with flow cytometry to study the clinically relevant HIV latency, we used the JLat 9.2 cell line. Latent HIV activation is much more difficult to achieve in JLat 9.2 cells compared to JLat 10.6. Therefore, the characterization of HIV activation in JLat 9.2 cells is important as it more closely mimics the behavior of HIV reservoirs in vivo, which are difficult to activate [32].

Due to the inherently low levels of HIV activation in JLat 9.2 cells, we determined the optimal stimulation period for GFP expression in JLat 9.2 cells over 24 h. We observed only 2% GFP+ JLat 9.2 cells at 12 h post stimulation, which increased to 12% after 24 h (Supplementary Materials SI-3). In contrast, 46% of the stimulated JLat 10.6 cells were GFP+ at 12 h, increasing to 68% at 24 h (Supplementary Materials SI-3). Thus, not only does a lower proportion of the JLat 9.2 population become activated, but the time course of activation is also slower. As a result, we chose to stimulate the JLat 9.2 cells for 24 h prior to the DNP-NMR analysis. We did not extend the activation period beyond 24 h in order to minimize the incorporation of the ^15^N-enriched amino acids into proteins other than GFP or HIV. We also note the variability in the percentage of the JLat 10.6 GFP+ cells between experiments. This highlights the importance of combining flow cytometry and other reporting techniques with in-cell NMR techniques for the accurate assessment of NMR spectra.

**Figure 4:**
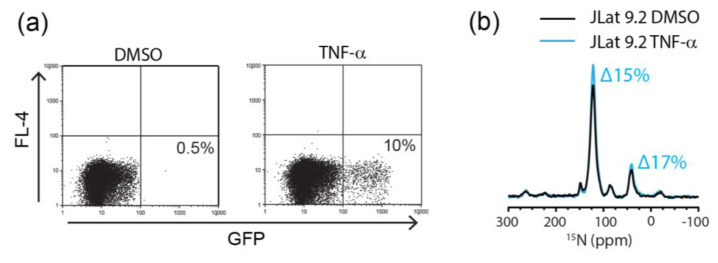
Characterization of the HIV production in JLat 9.2 cells. (**a**) Flow cytometry of the JLat 9.2 cells treated with 1% DMSO (vehicle control) or TNF-α. Plots are gated on live cells with the total percentage of GFP+ cells indicated in each plot (bottom right quadrant). (**b**) ^15^N CPMAS-DNP spectra of 1% DMSO (black line)- or TNF-α (blue line)-treated ^15^N-labeled JLat 9.2 cells. Spectra were acquired at 7.05 T and 95 K with 197.674 GHz microwave irradiation, 4.5 kHz MAS and 2048 transients.

We recorded the ^15^N cross polarization magic angle spinning (CPMAS)-DNP spectra of the JLat 9.2 cells stimulated with TNF-α for 24 h and observed a 15% and 17% increase in the intensity of the amide and amine resonances, respectively (Figure 4b). This correlates reasonably well with the flow cytometry data, where 10% of the stimulated JLat 9.2 cells were GFP+ (Figure 4a). Furthermore, the observation of this increase demonstrates the excellent sensitivity of the DNP-NMR to detect small populations of latently activated JLat cells within a heterogenous sample. However, the correlation between the percent GFP+ cells and the ^15^N signal intensity is therefore not accurate enough to allow the use of ^15^N amide resonance intensity as a proxy for the GFP/HIV expression alone. Additional factors must therefore influence the ^15^N signal intensity in the activated JLat cells.

### 2.3. Flow Cytometric Sorting Improves Cell Homogeneity

**Figure 5:**
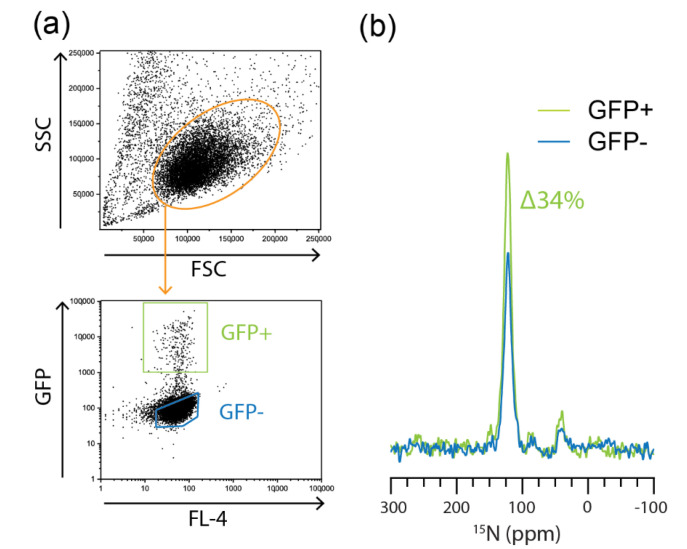
Increased cellular homogeneity using flow cytometric sorting. (**a**) Flow cytometry gating strategy for the GFP sorting of TNF-α-activated JLat 9.2 cells. Side scatter (SSC) and forward scatter (FSC) profiles were used to gate live cells. (**b**) ^15^N DNP-NMR spectra of 3 × 10^6^ GFP+ and GFP− cells.

The contribution of background T cell activation to ^15^N resonance intensity is difficult to ascertain when comparing non-stimulated with stimulated JLat cells. To do this requires the separation of GFP+ TNF-α stimulated JLat cells from their GFP− counterparts. We achieved this by using flow cytometry to sort GFP+ cells and GFP− cells from a heterogenous population of TNF-α-stimulated JLat 9.2 cells. Due to the limited sorting capacity of flow cytometers, only 3.6 × 10^6^ GFP+ and GFP− cells could be collected. However, we could still resolve a 34% increase in ^15^N-amide resonance intensity in the GFP-enriched samples compared to GFP− samples (Figure 5). This is a further demonstration that the increased signal intensity is due to the expression of HIV and associated GFP as the incorporation of flow cytometric sorting normalizes for any additional protein production due to stimulation. Flow cytometric sorting, in general, yields good cellular homogeneity but at the expense of total cellular mass. Alternative methods for bulk cellular purification such as antibody-based sorting could be employed and will be an important step for the analysis of primary cells and clinical samples where the occurrence of virally infected cells is very low.

The difference in the signal intensity between GFP+ and GFP− was expected to be much larger than what was observed. Given the 15% increase in the ^15^N signal intensity of the stimulated JLat 9.2 cells compared to the unstimulated cells, which represents nearly a 1% increase in the ^15^N signal intensity per 1% GFP expression, we expected close to a 100% increase in the ^15^N signal intensity of the sorted GFP+ JLat 9.2 cells compared to the sorted GFP− JLat 9.2 cells. This may be due to the upregulated basal protein production by TNF-α stimulation which partly cancels out the enhancements we would expect to see if the increase in protein content could be solely attributed to HIV and GFP. Furthermore, it is not clear how much protein needs to be produced to result in a 1% increase in signal intensity in this endogenous system.

The JLat MAS-DNP system is a promising new tool for the study of HIV virion production and latency reversal at the molecular level. The work presented here is the first demonstration of the DNP-enhanced solid-state NMR detection of endogenously produced HIV virions. The detection of increased amide resonance intensities by the MAS-DNP of HIV reactivated JLat cells provides an excellent model for future multi-dimensional studies of HIV virions within intact human cells. The benefits of multi-dimensional spectroscopy are the significant gains in data resolution through reduced spectral overlap with every additional dimension. This is a significant caveat for DNP-solid-state NMR as at cryogenic temperatures, molecular motion that is present at ambient temperatures is frozen out and can manifest as line broadening in DNP spectra, greatly reducing sensitivity and resolution. In addition, in-cell experiments further challenge NMR sensitivity due to the low concentration of spins of interest. Further gains in DNP and NMR sensitivity through technological developments will significantly improve the implementation of multi-dimensional NMR analysis to proteins at physiological concentrations (nano and micromolar concentrations) and in the heterogeneous environment of intact cells.

The combination of cell sorting and purification strategies provides a means for addressing the challenge of cellular heterogeneity for in-cell NMR studies to improve sensitivity and sample normalization. We hope to foster the integration of new developments in NMR methods and instrumentation with rigorous and well established biological techniques. Further development of our integrated approach could open up more biologically relevant systems to the in situ study at the atomic level.

## 3. Materials and Methods

### 3.1. Cell Lines and Cell Culture

JLat 10.6 and JLat 9.2 cells were obtained from the NIH AIDS Reagent Program, Division of AIDS, National Institute of Allergy and Infectious Diseases (catalog #1340), NIH.: J-Lat Full Length Cells from Dr. Eric Verdin [43]. This cell line has been previously described [42,43]. Briefly, the Jurkat T cells were infected with HIV-R7/E-/GFP retroviral vector and displayed no virus production under basal growth conditions. These cells express the HIV proteins Gag-pol, vpu, vpr, vif, rev and tat but not nef or env (gp120/gp41). They produce viral particles that are shed from the cells but are non-infectious due the absence of env. Standard Jurkat T cells were purchased from the ATCC. All three cell lines were continuously cultured in Roswell Park Memorial Institute (RPMI) 1640 medium (Gibco; Thermo Fisher Scientific, Waltham, MA, USA) supplemented with; 10% heat-inactivated fetal bovine serum (Gibco; Thermo Fisher Scientific, Waltham, MA, USA); l-glutamine 4 mM (Gibco; Thermo Fisher Scientific, Waltham, MA, USA); 100 U/mL penicillin 100 U/mL streptomycin (Gibco, USA) and 0.5 mM sodium pyruvate (Gibco; Thermo Fisher Scientific, Waltham, MA, USA) to make complete RPMI (cRPMI) 1640 medium. Isotopic labeling was performed using in-house cRPMI ((^15^N-G,L,K,Q,E,T,V,P)-RPMI) following the Thermo Fisher Scientific recipe [53] containing (^15^n-amide Glycine (10 mg/mL), Leucine (50 mg/mL), Lysine (40 mg/mL), Glutamine (300 mg/mL), Glutamate (20 mg/mL), Threonine (20 mg/mL), Valine (20 mg/mL), Proline (20 mg/mL)) and fetal bovine serum dialyzed against PBS to remove unlabeled amino acids. All amino acids were purchased from Cambridge Isotope Laboratories (Tewksbury, MA, USA).

### 3.2. HIV Reactivation

JLat cells in exponential growth were resuspended in ^15^N-(G,L,K,Q,E,T,V,P)-cRPMI at 3 × 10^6^ cells/mL and treated with 1% DMSO (*v*/*v*) or 10 ng/mL Tumor Necrosis Factor α (TNF-α) (Sigma-Aldrich, Saint Louis, MO, USA) (with 1% DMSO). The cells were incubated at 37 °C for 12–24 h with 5% CO_2_ in a humidified incubator.

### 3.3. Sample Preparation

After stimulation, the JLat cells were prepared for NMR by pelleting 36 × 10^6^ cells at 170× *g* for 5 min and then washing with 10 mL 1 × PBS to remove the cell culture media, unused isotopes and secreted proteins. The cell pellet (30 µL volume) was then resuspended in 30 µL of DNP solution containing 1 × PBS, 10% DMSO (*v*/*v*) and 20 mM AMUPol (Cgiving a final AMUPol concentration of 10 mM. The cell suspension was funneled directly into a Y_2_O_3_-stabilized ZrO_2_ NMR rotor without further washing at 800× *g* for 20 s using custom-made Teflon funnels and frozen immediately in liquid nitrogen to halt the cellular reduction of AMUPol. The total packing time, defined as the time from the addition of AMUPol (to the cell pellet) to the freezing of the sample in liquid nitrogen, was less than 2 min.

Samples for the flow cytometry were prepared by taking 100 μL aliquots of isotopically-labeled JLat cells (~100,000 cells) and then fixing in 2% formaldehyde overnight at 4 °C prior to flow cytometric analysis.

### 3.4. NMR Data Collection

All experiments were performed on a 7.05 T magnet at 95 K with Larmor frequencies of ^1^H = 300.184 MHz and ^15^N = 30.179 MHz, using a custom-built, 4-channel, transmission line NMR probe housing a 3.2 mm outer diameter rotor and controlled via a Redstone spectrometer (Tecmag Inc., Houston, TX, USA). DNP was performed with a custom-built 198 GHz gyrotron outputting 40 W microwave irradiation at a frequency of 197.674 GHz. Incident power at the sample was approximately 7 W. A rotor-synchronized, echo-detected CPMAS sequence from ^1^H to ^15^N nuclei was used to record all the ^15^N spectra at 4.5 kHz MAS. A Hartmann–Hahn contact time of 1 ms was used with Rabi frequencies of ^1^H = 83 kHz and ^15^N = 77 kHz. Two Pulse Phase Modulation (TPPM) ^1^H decoupling was employed with ^1^H = 83 kHz. Magnetization was saturated using a pulse train on the ^1^H and ^15^N channels before the DNP polarization period (duration of 3 s). A total of 2048 transients were acquired per sample. The data were processed in TNMR v3.2.8 (Tecmag Inc., Houston, TX, USA). Peaks were fit using DMFit*#20200306* (CEMHTI CNRS, Orleans, France) [54] to determine intensities.

### 3.5. EPR Analysis

JLat cells (12 × 10^6^ cells) were prepared as described previously. Cell pellets (10 μL) were resuspended in 10 μL of 1 × PBS with 10% DMSO and 20 mM AMUPol and centrifuged into EPR tubes at 800× *g* for 20 s. Excess supernatant was removed and the sample was frozen immediately in liquid nitrogen with a total packing time less than 3 min, to halt any reduction of AMUPol prior to EPR analysis. For analysis, the capillary tube was thawed to room temperature then immediately inserted into a 5 mm NMR tube to fit the cavity of a 9.4 GHz EPR spectrometer (JEOL-JES-FA 100). Continuous wave EPR spectra was acquired at 9.4 GHz with 2 mW microwave power and a sweep time of 30 s (0.1 s time constant) using a modulation frequency of 100 kHz over 0.1 mT and an amplitude of 50. All spectra were acquired at room temperature for 30 min.

### 3.6. Flow Cytometry Analysis

GFP fluorescence of fixed, isotopically labeled JLat 10.6 and JLat 9.2 cells was analyzed using a FACScan flow cytometer (BD Biosciences, Franklin Lakes, NJ, USA). Live cells were gated based on forward (FSC) and side scatter (SSC) profiles. The data were analyzed using Flowing Software v2.5.1 (Perttu Terho). For the GFP time course analyses, 1 × 10^6^ cells were plated at 3 × 10^6^/mL and treated with 10 ng/mL TNF-α. The samples were collected at 0, 12, and 24 h and analyzed by flow cytometry.

For the cell-sorting experiments, 100 × 10^6^ JLat 9.2 cells were plated at 3 × 10^6^/mL in ^15^N-(G,L,K,Q,E,T,V,P)-cRPMI media and treated with 10 ng/mL TNF-α for 24 h. Cells were then sorted on a FACSAria flow cytometer (BD Biosciences). Live cells were gated based on forward and side scatter profiles as depicted in Figure 5. After sorting, the cells were counted, and 3.6 × 10^6^ GFP+ and GFP− cells were packed into 3.2 mm Y_2_O_3_-stabilized ZrO_2_ rotors as described above.

## Supplementary Materials

Supplementary Materials can be found at https://www.mdpi.com/1422-0067/21/13/4649/s1.

## Abbreviations

cRPMI	Complete Roswell Park Memorial Institute
DNP	Dynamic nuclear polarizations
FACS	Fluorescence-activated cell sorting
HIV	Human immunodeficiency virus
JLat	Jurkat latency model
NMR	Nuclear magnetic resonance
PKC	Protein kinase C
TNF-α	Tumor necrosis factor alpha
DMSO	Dimethyl sulfoxide
GFP	Green fluorescent protein
MAS	Magic angle spinning
HEK	Human embryonic kidney cells
PBS	Phosphate buffered saline
CPMAS	Cross polarization magic angle spinning
