# Supplementary material for: In Situ Detection of Endogenous HIV Activation by Dynamic Nuclear Polarization NMR and Flow Cytometry
Source: Int J Mol Sci. 2020 Jun 30;21(13):4649. doi: 10.3390/ijms21134649 (PMC7369949; doi:10.3390/ijms21134649)
Supplement: Supplementary file 1 [file ijms-21-04649-s001.pdf]

Supplementary Material

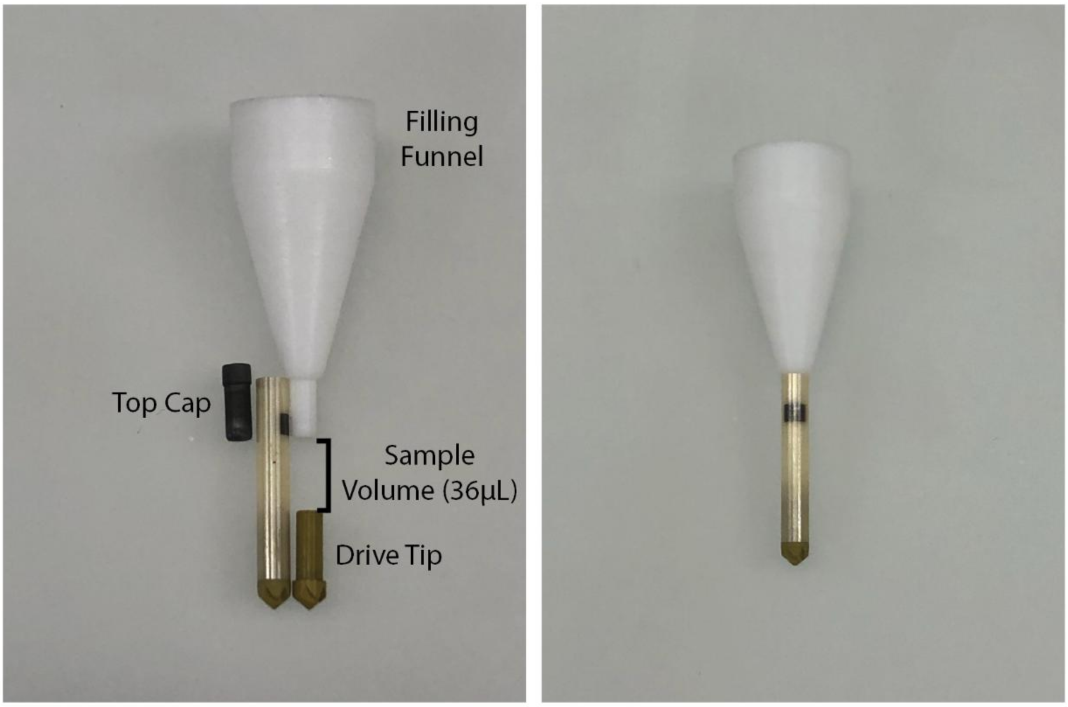

SI-1. Custom made filling funnels that control the rotor filling volume. Images show the arrangement of drive tip and top cap relative to the rotor volume and compared to the volume filled by the funnel. The rotor assembled with the funnel is shown on the right.

Isotopically labeled amino acids used in this study

Protein	UniProt ID	#aa	#Gly	#Leu	#Lys	#Glu	#Gln	#Thr	#Val	#Pro
**Gagpol**	P04585	1435	108	110	122	95	107	88	96	77
**Rev**	P04618	116	10	13	2	7	11	8	5	10
**Tat**	P04610	86	6	3	9	6	3	8	3	11
**Vif**	P69723	192	13	16	14	8	8	10	12	10
**Vpr**	P69726	96	8	9	1	7	10	4	3	4
**Vpu**	P05919	81	4	6	2	2	8	0	10	3
**GFP**	P42212	238	22	19	20	8	16	15	17	10

Total amide ^{15}N labels: 1217

Total amide ^{15}N labels from HIV associated proteins: 1090/1217 = 89%

Total amide ^{15}N labels from GPF = 127/1217= 11%

SI-2

Amino acid composition of HIV and GFP proteins. Sequence composition was obtained by taking the uniprot sequences (IDs given) and calculating amino acid composition using ProtParam Tool from (https://web.expasy.org/protparam/). ^{15}N was only incorporated into the free amine associated with the amide nitrogen (side chain nitrogens are natural abundance) and so the total number of labels is equal to the total number of amino acids for which ^{15}N was incorporated.

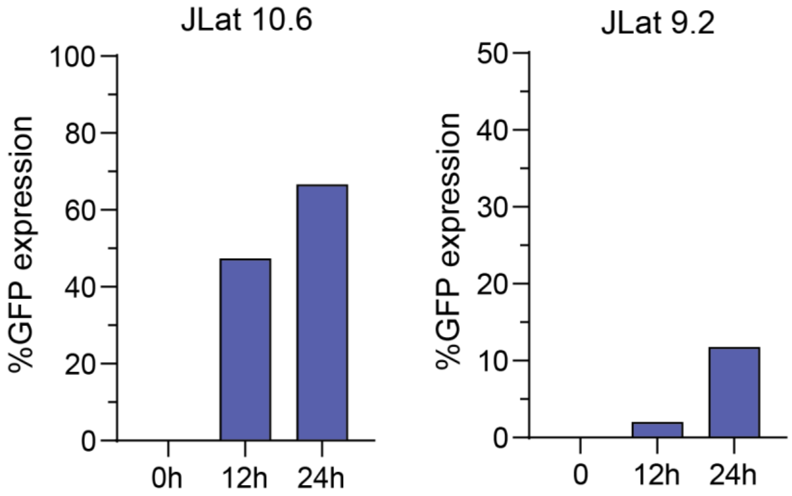

SI-3. Time course of GFP expression in JLat 10.6 and JLat 9.2 cells following TNF-α activation analyzed by flow cytometry
